# Mechanical Behavior of a Composite Lightweight Slab, Consisting of a Laminated Wooden Joist and Ecological Mortar

**DOI:** 10.3390/ma13112575

**Published:** 2020-06-05

**Authors:** Carmelo Muñoz-Ruiperez, Francisco Fiol Oliván, Verónica Calderón Carpintero, Isabel Santamaría-Vicario, Ángel Rodríguez Sáiz

**Affiliations:** Department of Construction, University of Burgos, Calle Villadiego s/n, 09001 Burgos, Spain; cmruip@ubu.es (C.M.-R.); ffiol@ubu.es (F.F.O.); vcalderon@ubu.es (V.C.C.); isantamaria@ubu.es (I.S.-V.)

**Keywords:** wooden joist, mechanical behavior, full-scale flexural test, lightweight mortar, expanded clay, recycled concrete aggregates (RCA), recycled mixed aggregates (RMA)

## Abstract

The investigation reported in this paper is an evaluation of the mechanical behavior of full-scale ecological mortar slabs manufactured with a mixture of expanded clay and recycled concrete aggregates. The composite mortars form a compressive layer over laminated wooden joists to form a single construction unit. To do so, full-scale flexural tests are conducted of the composite laminated wood-ecological mortar slabs with different types of mortar designs: reference mortar (MR), lightweight mortar dosed with recycled concrete aggregates (MLC), and lightweight mortar dosed with recycled mixed aggregates (MLM). The test results showed that the mortar forming the compression layer and the laminated wooden joists worked in unison and withstood a higher maximum failure load under flexion than the failure load of the wooden joists in isolation. Moreover, the laboratory test results were compared with the simulated values of the theoretical model, generated in accordance with the technical specifications for structural calculations contained in the Spanish building code, and with the results calculated by a computer software package. From the analysis of the results of the calculation methods and the full-scale laboratory test results, it was concluded that the safety margin yielded by the calculations validated the use of those methods on this type of composite slab. In this way, a strong mixed wood–mortar slab was designed, contributing little dead-load to the building structure and its manufacture with recycled aggregate, also contributes to the circular economy of construction materials.

## 1. Introduction

At present, new urbanistic tendencies are oriented towards the recovery of the built environment, promoting the rehabilitation of classic buildings, historic city centers, and old degraded housing units [1,2,3]. European cities conserve buildings constructed between the 17th and the 18th century, with beautiful façades and interiors of incalculable historic value that are in need of conservation [4,5,6,7].

The horizontal structures of most of these historic buildings are made of wood, a noble material although sensitive to the maintenance conditions of the building, and subjected to working stresses close to their acceptable limitations. The old wooden structures often require reinforcement structures, because they present structural damage and are in need of conservation, due to humidity, xylophages, and excessive deformability under excessive loads [8,9,10,11,12].

Traditionally, various constructive solutions have been applied, to address these issues, such as the installation of lightly reinforced concrete floors over the pre-existing and previously treated structure, with no type of connection between either levels. An arrangement that means that the two structural elements constituted in that way will function independently of each other, in such a way that the carrying capacity and the rigidity of the building are not increased [13,14,15]. The advantage of this solution is that it permits the redistribution of loads between the primary and the secondary beams, but considerably increases the overall weight, which can in some cases exceed the efficacy of the reinforcement. Other constructive solutions attach joists to the concrete or mortar floor, so that the connections absorb the shear stresses between both materials, ensuring that they work together in unison [16,17,18,19].

The use of wooden–concrete structures attached by connectors is a relatively recent technique, the first studies on which by Richart and Williams [20] date back to 1943. Moreover, the use of composite structures has been investigated in various studies, in order to understand their behavior better and to develop acceptable technical solutions for their use in construction, centering on their global behavior, experimental analysis, and wood–concrete connector systems [20,21,22,23,24].

On the one hand, a challenge for our society today is to put into practice development strategies that are respectful of the environment and natural settings, and compatible with a sustainable, circular economy. The new social initiatives for environmental defense require the substitution of lineal models of development where excessive quantities of resources are used, for circular economies, in which the waste generated in industrial processes is recovered as a raw material [25,26], thereby avoiding the over-exploitation of natural resources [27,28].

The economic activity of the construction industry is among those with the highest impact on the natural environment, because both the manufacture of materials and the construction of infrastructures and buildings consume large quantities of resources and produce significant amounts of waste, as well as modifying the landscape, all of which directly affects the natural environment [29].

The manufacture of construction materials from waste co-products generated in construction processes represents an alternative to the use of traditional materials. Oriented towards the design of new ecological materials and using recovered construction waste, the investigations that have been developed over recent years have advanced considerably [30,31,32,33]. Likewise, the use of recovered waste has been considered for the manufacture of structural materials such as concrete, either separately or jointly with other aggregates, one example of which is lightweight ceramic materials [34,35,36].

In preceding works, a complete characterization of mortars designed with construction waste and expanded clay was presented, describing a material with good mechanical performance and acceptable durability levels over time. The mortar design presented good mechanical behavior, both flexural and under compression, which makes it ideal for use in lightweight structural frameworks [37,38].

The investigation reported in this paper represents an attempt to respond to the structural rehabilitation of historic buildings. To do so, a mixed slab was formed of ribs of laminated wooden joists and a compression zone, consisting of an ecological mortar manufactured with recycled aggregate and expanded clay aggregate.

Subsequently, the mechanical behavior of the mixed slab was studied under working stress, assessing the strength of the composite wood–mortar slab. Its assessment was through a comparison with the theoretical simulation model, using the real values obtained from the failure tests, and verification of the safety reserve that guarantees system stability.

The final objective is to achieve a strong slab, with a compression zone manufactured with a sustainable mortar containing recycled aggregates that contributes less additional load to the existing building structure.

## 2. Materials and Methods

### 2.1. Cement Mortars

The cement mortars manufactured with construction waste and expanded clay, used as a compression layer in the laminated wood–mortar construction component design have previously been characterized in earlier investigations [37,38].

#### 2.1.1. Raw Materials

**Figure 1:**
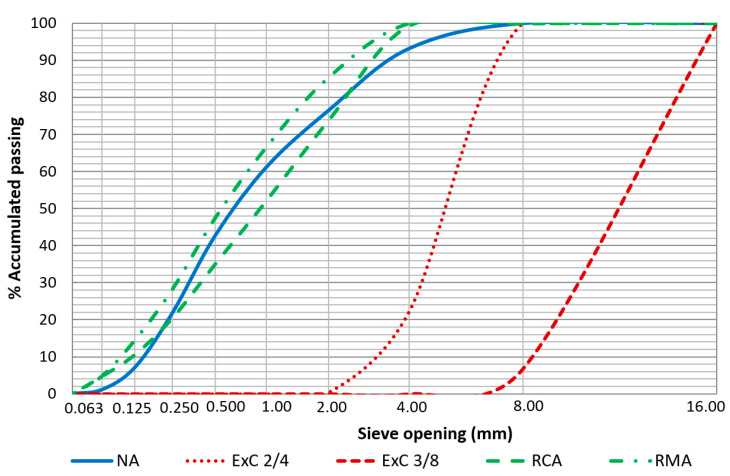
Particle size distribution of raw materials.

- Binder: A CEM 1 42.5 R cement was used, in accordance with the technical specifications of standard EN 413-1:2011 [39], supplied by the firm Cementos Portland Valderribas (Navarra, Spain), and manufactured at its Mataporquera factory (Cantabria, Spain). It has a density of 3160 kg/m^3^ and a Blaine specific surface of 340 m^2^/kg. Its chemical composition, obtained by X-ray fluorescence was mainly CaO (60.4%) and SiO_2_ (21.3%).
- Natural aggregates (NA): washed natural sand from an open-cast quarry situated in the locality of Cubillo del Campo (Burgos, Spain). Composed of SiO_2_ (95.29%), its particle density was 2640 kg/m^3^, meeting the specifications of standard EN 1097-6 [40]. The granulometric curves of the aggregate employed in the mixtures are shown in Figure 1.
- Expanded clay (ExC): the expanded clay used in the mixtures was supplied by the firm Argex—Argila Expandida S.A. (Bustos, Portugal). Two types of commercial granulometries were employed under the following commercial brands: Argex^®^ 2-4 (ExC2/4) with an apparent dry density of 358 kg/m^3^, and Argex^®^ 3-8F (ExC3/8) with an apparent dry density of 300 kg/m^3^.
- Recycled concrete aggregate (RCA): from the fragmentation of defective pre-fabricated concrete components manufactured by the firm “Artepref” (Aranda de Duero, Burgos, Spain), with a particle density of 2400 kg/m^3^. Chemical composition SiO_2_ (56.53%) and CaO (37.40%).
- Recycled mixed aggregate (RMA): this aggregate is from the recycling plant of the waste transport and management firm “Iglecar S.L.” (Burgos, Spain). Its particle density was 2450 kg/m^3^ and its chemical composition was SiO_2_ (67.66%), CaO (22.08%) and Al_2_O_3_ (5.02%).

#### 2.1.2. Mortar Mixtures

Three different mortar dosages were used for manufacturing the compression layer of the slab framework:

- Reference mortar (MR): prepared with Natural Aggregate (NA), with a 1:4:w (cement: aggregate: water) dosage by weight of raw materials.
- Lightweight mortar MLC: 75% of the NA was substituted in this mortar by expanded clay (ExC), specifically, 56.25% by ExC2/4 and 18.75% by ExC3/8. The remaining 25% was substituted by recycled concrete aggregates (RCA).
- Lightweight mortars MLM: 75% of the NA was substituted in this mortar by expanded clay (ExC), specifically, 56.25% by ExC2/4 and 18.75% by ExC3/8. The remaining 25% was substituted by recycled mixed aggregates (RMA).

The relation of components by weight of the ecological mortar designs are shown in Table 1.

The mortars designed with recovered construction waste and expanded clay have been studied in earlier investigations [37,38], in accordance with the specifications of the European standard. Their characteristics, both in the fresh and in the hardened state, are shown in Table 2.

### 2.2. Laminated Wood

**Figure 2:**
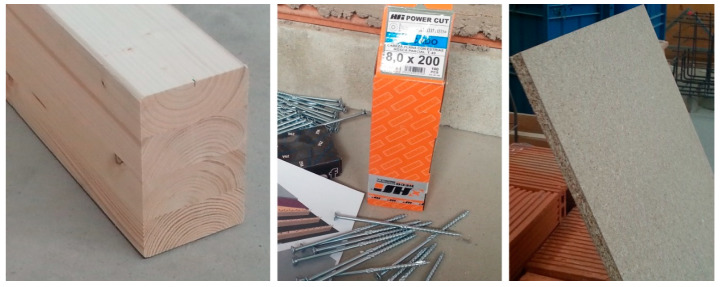
Wooden slab framework and connectors (left to right: joist, connectors, and board).

The wood used for the manufacture of the composite laminated wood-ecological mortar slab was supplied by the firm “Arte y Madera, S.A.” (Burgos, Spain). The slab consisted of the following materials (Figure 2):

- Wooden laminated (Gulam) joists of Douglas Fir GL24c with a width and height of (100 × 160) mm^2^ and a length of 1500 mm. The Gulam joist is composed of five even layers, each with a thickness of 32 mm. Their mechanical properties and characteristics are shown in Table 3.
- Agglomerated wooden board with dimensions of (500 × 1500) mm^2^ and a thickness of 15 mm. The board is used as a sort of lost formwork for pouring the mortar. Its characteristics were not computed in the theoretical calculations.
- Connectors between the joist and the construction mortar layer: bichromate-plated self-tapping flat-topped round screws with a diameter of 8 mm and a length of 200 mm.

### 2.3. Composite Framework: Wood–Mortar Section

**Figure 3:**
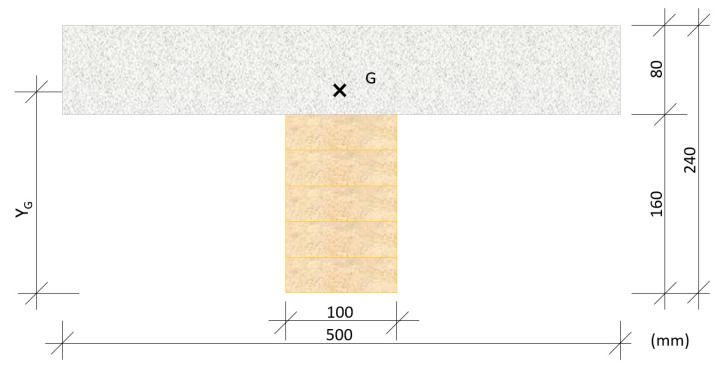
Section of the constructive component.

The composite slab section design in this investigation consisted of a laminated wooden (Gulam) joist, next to a compression layer of cement mortar manufactured with recovered construction waste and expanded clay, as shown in Figure 3.

where, in Figure 3:

Y_G_—Distance from the neutral fiber of the wood (Gulam) to the center of mass of the composite section.

G—The cross represents the center of mass of the composite section.

The effective rigidity, (n), of the unified section of the constructive component consisting of laminated wood–mortar was calculated by the following expression, as a function of their respective moduli of elasticity, *E*.

(1) $$n=\frac{E_{m}}{E_{c}}$$

where,

*E_m_*—Elasticity modulus of the wood (see Table 3)
*E_c_*—Elasticity modulus of the mortar (Equation (2))

The elasticity modulus (*E_m_*) of the mortar was calculated with the specifications from the Structural Concrete standard EHE-08 [49] as a function of the average compression strength obtained from the tests, with the expression

(2) $$E_{c}=8500\sqrt[{3}]{f_{cm}}$$

where,

*f_cm_* is the average compression strength of the mortar (*f_cm_* = 35 MPa for the MR mortar, and an average value of *f_cm_* = 17 MPa for the MLC and the MLM mortars).

The mechanical properties for the study of the composite section, considering the effective rigidity of the laminated wood–cement mortar composite are shown in Table 4.

The objective is to determine the maximum theoretical failure load for the constructive laminated wood–mortar component with each mortar as a compression layer, in order to compare it with the failure load from the full-scale test. Likewise, the stress state in the upper fiber of the (mortar) construction component and in the lower fiber (laminated wood joist) will be determined.

## 3. Methodology

### 3.1. Preparation of Specimens

**Figure 4:**
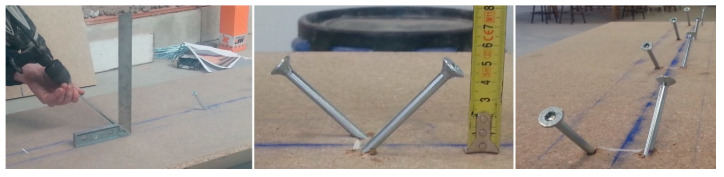
Placing the connectors.

**Figure 5:**
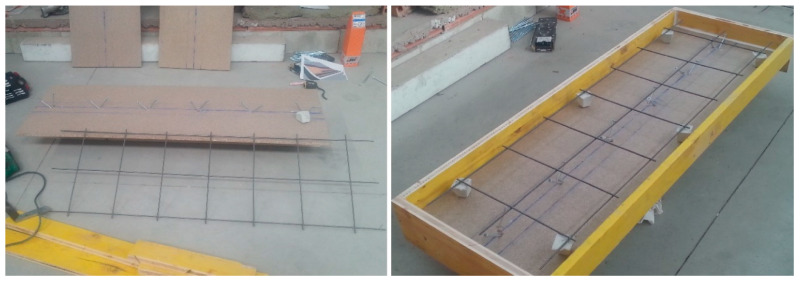
Final appearance of the mesh and formwork of the constructive unit.

Six constructive units were prepared for the industrial test in the laboratory, two for each of the mortars used as the compression layer (MR, MLC, and MLM), as per the following process, in accordance with the scheme shown in Figure 3:

- In the first place, the agglomerated wooden board was attached to the laminated wooden joist of Douglas Fir by six pairs of connectors (in total, 12 metal screws), at intervals of 26 cm from pair to pair, positioned at an angle of 45° and at an approximate height of 50 mm over the upper surface of the board (Figure 4).
- Subsequently, boarding was positioned around the perimeter that functioned as shuttering for the compression layer of mortar. This formwork consisted of three-layer cross-laminated wooden boards.
- As reinforcement, an electro-welded mesh formed of 5 mm diameter bars with a mesh span of (200 × 300) mm^2^ was used. With the objective of guaranteeing an upper cover of 25 mm, the reinforcement was supported upon small mortar blocks (Figure 5).

Having positioned the reinforcements in place, the mortar designed with recovered construction waste and expanded clay was mixed. The components that were used and their dosages are shown in Table 5.

A Mark T-Mech electric mixer with a capacity of 70 L was used for mixing the components, introducing in the first place the aggregates and 50% of the required volume of water, mixing the mass of concrete for 2 min. Subsequently, the cement was added and the remaining 50% of the water, mixing for 2 min to achieve an even mixture.

**Figure 6:**
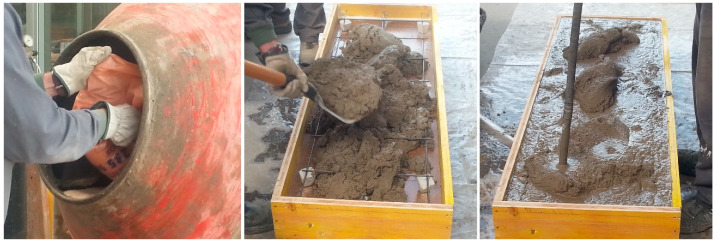
Manufacture mortar (**left**), placing of the mortar (**center**) and mortar compactation (**right**).

The mortar compression layer was done by pouring two 4-cm-thick mortar layers, compacted with a water vibrator. The excess mortar was removed using a metallic rod, levelling off the mixture at the level of the upper board of the formwork, leaving a smooth, flat and even top surface (Figure 6).

The six construction slab components remained in the laboratory for 28 days at a temperature of 20 °C and a relative humidity of 50%. The mortar was covered with plastic for the first seven days of curing, to minimize water evaporation. Over the first few days, water was softly pulverized over the surface to humidify it, thereby avoiding loss of mix water due to the increased hydration heat, in order to ensure proper setting and hardening of the mortar. After 7 days, the plastic was removed and the samples remained in the laboratory until the 28 days of curing was over.

### 3.2. Full-Scale Flexural Test of the Composite Slab

**Figure 7:**
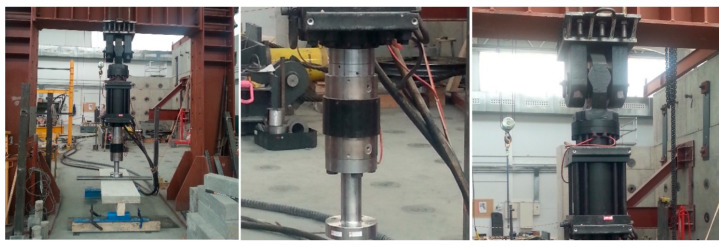
1000 kN hydraulic press (**left**). Detail of actuator (**center**) and of transducer (**right**).

Following 28 days, the mortar had properly set and hardened and the flexural test could be performed using a hydraulic press in the laboratory with a load capacity of 100 t, connected to a five-channel electronic measurement unit. The press is an MTS brand, model 201.70 HF, with the following characteristics: a tension force of 965 kN and a compression force of 1460 kN. Equipped with an MTS transducer model 661.31F-01, it had a capacity of 1000 kN (Figure 7).

It can apply continuous and variable loads, with the objective of achieving a uniform displacement of the transducer. The application of a variable downward load in the test was decided upon at a velocity of 0.01 mm/s.

**Figure 8:**
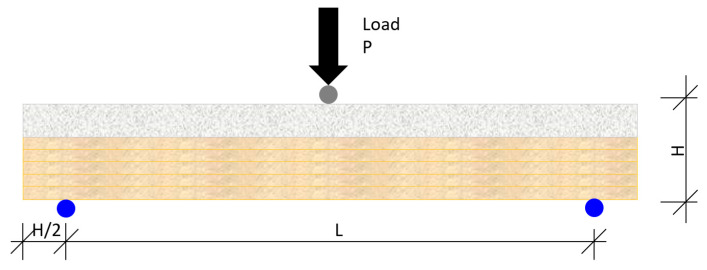
Diagram of the flexural failure test.

**Figure 9:**
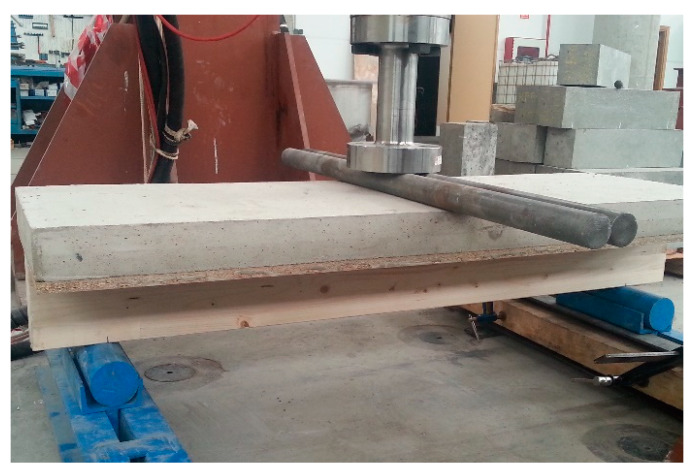
Flexural failure test set up.

The process was managed with the MTS Flex Test GT digital controller [50], which displays information from the force transducer that controls the force applied through the hydraulic piston, and records both force and displacement. The flexural failure test was performed in accordance with the diagram that is shown in Figure 8 and Figure 9:

where,

P—Force applied through the transducer
H—Full-scale height of the slab component
L—Length between supports

### 3.3. Analytical Models

The analytical models that will be employed, in application of the current norms in the European Union (EuroCode 5) [51] and in Spain (Código Técnico de la Edificación Documento Básico SE-M Seguridad en Madera) [52], are explained in the following sections [53].

The specific loading hypotheses are, on the one hand, the dead loads corresponding to the weight of the materials, and on the other, the variable load applied by the hydraulic piston up until the failure limit, as described in Section 3. Subsequently, the ultimate limit state criteria that corresponded to the composite section will be applied, in so far as it refers to the fatigue failure of sections subjected to stress orientation, along the main directions. These verifications fundamentally correspond to shear forces between the section of the wooden joist and the mortar in the compression layer through the connection, so that the collaboration was effective as a mortar-wood joist to protect against fatigue (breakage), taking the section subjected to simple flexion.

#### 3.3.1. Ultimate Fatigue Limit State of the Sections Subjected to Shear Forces—Justification of the Union of the Laminated Wood–Mortar Composite Section

**Figure 10:**
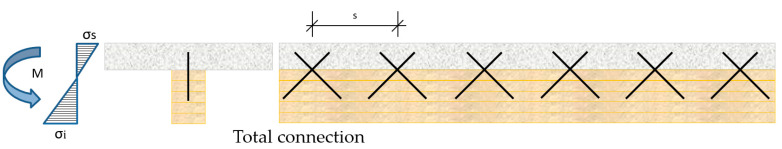
Diagram of wooden joist-compression layer connections.

The theoretical requirement for collaboration of the mortar layer in the laminated wood-ecological mortar construction unit design is that the screws or connectors will prevent the displacement of the head of the joist, for which reason it was necessary to arrange them at an angle, as shown in Figure 4 and Figure 10.

This arrangement guarantees that the collaboration between both materials laminated wood-ecological mortar is effective and the total height of the slab section will be the sum of the heights (height of the laminated wood and of the mortar). This design principle will ensure that the stress forces are jointly shared, so that the neutral fiber of the constructive unit that is designed will be optimized.

The mathematical model recommended by EuroCode 5 was applied for the study of the shear force generated at the laminated wood–mortar interface [51]

(3) $$R_{d}<\varphi_{d}$$

where,

$R_{d}\;$—maximum shear force applied to the wood–mortar joint
$\varphi_{d}$—maximum shear strain that the vertical connection will withstand based on connectors in the composite section

The following expression was adopted, in order to calculate $\varphi_{d}$

(4) $$\varphi_{d}=n\;0.78\;d^{2}\frac{\sqrt{\frac{f_{hd}\times f_{yd}}{1.05}}}{s}$$

where,

$f_{hd}$—calculated crushing strength of the wood (16 N/mm^2^)
$f_{yd}$—elastic limit of steel connector reduced by a reduction coefficient of 1.05
n—number of connectors per section
d—diameter of the connectors (mm)
s—interval between planes of connection (mm)

The calculated value of the shear force, $R_{d}$, on the wood–mortar slab was calculated with the expression

(5) $$R_{d}=\frac{0.8\left(V_{pp}+V_{q}\right)}{0.90H}$$

where,

*V_pp_*—shear strength of composite slab
*V_q_*—shear force loading
H—section height

The result of the application of these formulas will determine the suitability of the collaboration between the composite wood–mortar sections.

#### 3.3.2. Ultimate Fatigue Limit State of the Section Subjected to Oriented Stress along the Main Directions—Simple Flexion

It must be ensured that the calculated stress forces of the loading ($\sigma_{md}$) will be less than the ultimate failure limit strength of the wood material ($f_{md}$), in order to guarantee the flexural strength of the laminated wood–mortar construction unit

(6) $$\sigma_{md}<f_{md}$$

The calculation hypotheses of the Technical Building Code-CTE-SE M [52], similar to those used by the Eurocode 5 [51] were considered, in order to calculate the theoretical ultimate limit states and their corresponding stresses.

The calculated strength under flexion of the wood, $f_{md}$, was calculated with the expression

(7) $$f_{md}=k_{mod}k_{h}\frac{f_{mk}}{\gamma_{m}}$$

where:

$k_{mod}$—is the modification factor in accordance with the class of duration of the load combination (in our case instantaneous load), the type of wood (laminated Gulam joist) and the class of service (service 1: temperature 20 ± 2 °C and relative humidity at 65%), in this case 0.60
$k_{h}$—coefficient that depends on the relative size of the section. According to the *CTE-SE M*, for rectangular sided GULAM joists under 600 mm, the following minimum values will be used

(8) $$k_{h}=\min\{\begin{array}{c} {\left(\frac{600}{h}\right)}^{0.1}\\ 1.1 \end{array}$$

where,

*h*—height of the side under flexion (mm)
$f_{mk}$—characteristic strength of the wood for GL24c (24 MPa)
$\gamma_{m}$—partial safety limit coefficient for the laminated wood, extraordinary situation 1.0

Substituting the earlier values in Equation (7), the following value of *f_md_* was given as

$$f_{md}=0.60\cdot 1.1\frac{24\;\mathrm{Mpa}}{1.0}=15.84\;\mathrm{Mpa}$$

The following expression was applied, to determine the calculated stress limit under flexion ($\sigma_{d}$) at the ultimate flexural moment of the section

(9) $$\sigma_{d}=\frac{M_{p}}{W_{d}}$$

where,

*M_p_*—ultimate flexural moment of the composite section
*W_d_*—strength modulus of the homogenized section (module W_i_Table 3)

The ultimate tensile stress under uniaxial solicitation, ($\sigma_{d})$, is equaled by the maximum admissible stress of the material, ($f_{md})$, in order to obtain the maximum load at the ultimate moment of failure. The tensile stress produced at the ultimate moment that is generated by the load is therefore equated with the maximum stress that the composite laminated wood–mortar section can withstand. In this way, it is possible to obtain the maximum ultimate load breaking the section

(10) $$\sigma_{d}=\frac{M_{p}}{W_{d}}=f_{md}=k_{mod}k_{h}\frac{f_{mk}}{\gamma_{m}}$$

Solving the above equation for $M_{p}$ yields

(11) $$M_{p}=W_{d}\frac{k_{mod}k_{h}f_{mk}}{\gamma_{m}}$$

The load, (P), at the ultimate moment, *M_p_*, of the section for a joist loaded mid-span and supported at both ends, was calculated with the expression

(12) $$M_{p}=\frac{PL}{4}$$

where,

P—load under flexion
L—length between supports (see Figure 8)

Equaling the ultimate moment of the composite section (11) with the load at the ultimate moment (12), yields

(13) $$M_{p}=W_{d}\frac{k_{mod}k_{h}f_{mk}}{\gamma_{m}}=\frac{PL}{4}$$

Solving the above equation for *P* yields

(14) $$P=\frac{4W_{d}k_{mod}k_{h}f_{mk}}{L\gamma_{m}}$$

The maximum stresses generated at the upper and lower fibers, both for the joist itself and the composite laminated wood–mortar section, were determined in the following way:

- Isolated wooden Gulam joist

The stress on the lower fiber, $\sigma_{i}$ (shear), and upper fiber, $\sigma_{s}$ (compression), were calculated with the following expression and compared with the maximum admissible stress, *f_mk_*, of the wood

(15) $$\sigma_{i}=\sigma_{s}=\frac{M_{p}}{W}\leq 24\;\mathrm{Mpa}$$

where,

1. *M_p_*—moment produced by the force P
2. W—strength modulus of the wooden section referring to h/2 (see Table 3)

**Figure 11:**
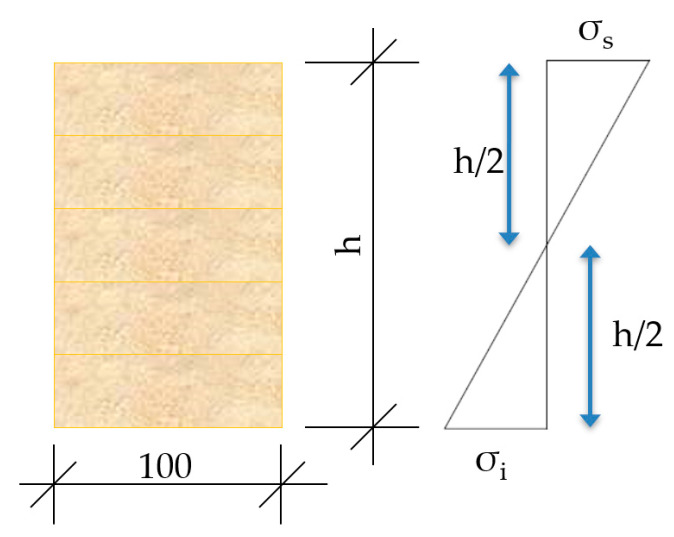
Stress state of an isolated joist.

A diagram is shown in Figure 11, of the stress state.

- Composite laminated wood–mortar section

The stresses that are exerted on the lower fiber, *σ_i_* (shear), of the composite section were compared with the maximum admissible stress, *f_mk_*, through the expression

(16) $$\sigma_{i}=\frac{M_{p}}{W_{i}}\leq 24\;\mathrm{Mpa}\;\left(f_{mk}\;wood\right)$$

where,

*σ_i_*—stress at lower fiber compared with the ultimate stress of the wood, *f_mk_*
*M_p_*—moment produced under solicitation (in this case strength *P*)
*W_i_*—strength modulus of the wooden section referring to Y_1_ (distance, Y_G_, to the lower fiber, see Table 4)

The stresses that were produced on the upper fiber, *σ_s_* (compression), of the composite section were compared with the maximum admissible stress, *f_k_*, of the mortars forming the compression layer, in accordance with the expression

(17) $$\sigma_{s}=\frac{M_{p}}{W_{s}}\leq 35\;\mathrm{Mpa}/17\;\mathrm{Mpa}\;(f_{k}mortar)$$

where,

*σ_s_*—upper fiber stress compared with the ultimate stress of the mortar under compression, *f_k_*, (MR/MLC/MLM)
*M_p_*—moment generated under solicitation, in this case force P
*W_s_*—strength modulus of the wooden section referring to Y_2_ (distance, Y_G_, to the upper fiber, see Table 4)

**Figure 12:**
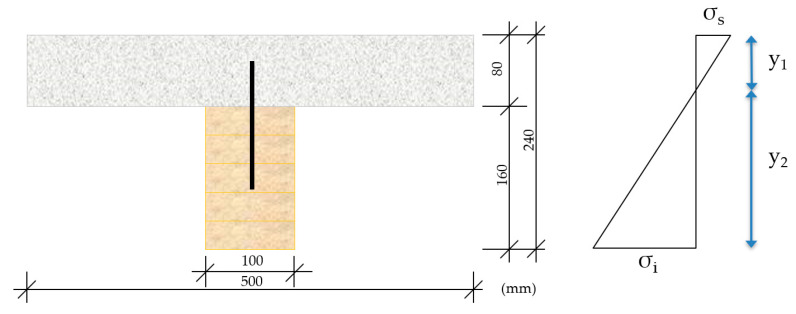
Stress state of the composite wood–mortar section.

In Figure 12, a diagram is shown of the stress state of the laminated wood–mortar compression layer construction unit.

#### 3.3.3. Theoretical Model, CYPE Program

Cype software, with the Cypecad plugin v2019.e [54] was used for modelling the design of the construction units. This program performs a three-dimensional spatial calculation with matrix rigidity methods, in which the connections between nodes are the elements that define the structure: pillar, joist, and joist header. In addition, the program can establish the deformation compatibility at all nodes considering all six degrees of freedom.

A static calculation was performed, for the purposes of obtaining the solicitations and displacements, at all loading states, considering a linear behavior of the material, in other words, a numerical calculation of the first order, for computerized computation.

**Figure 13:**
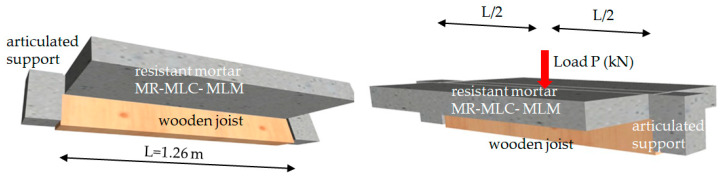
Discretization of the test model.

The program generates a mesh of bar-type elements sized 0.25 × 0.25 m (grid with node ports). A wooden joist was discretized and modelled for the design of the construction unit using the parameters listed in Table 1, and ecological mortar compression layers with compressive strengths, *f_ck_*, of 35 MPa (MR) and 17 MPa (MLC and MLM). The discretization of the test model may be seen in Figure 13.

## 4. Results

### 4.1. Full-Scale Flexural Test

Flexural failure tests were performed at the Large Infrastructures Laboratory of the Higher Polytechnic School of the University of Burgos, in order to test the behavior of the full-scale specimens.

Two specimens of the laminated wood-ecological mortar construction unit were tested for each slab that was constructed. The two joists of laminated wood and their performance were also separately analyzed, with the aim of establishing their influence on the strength capacity of the composite section.

**Figure 14:**
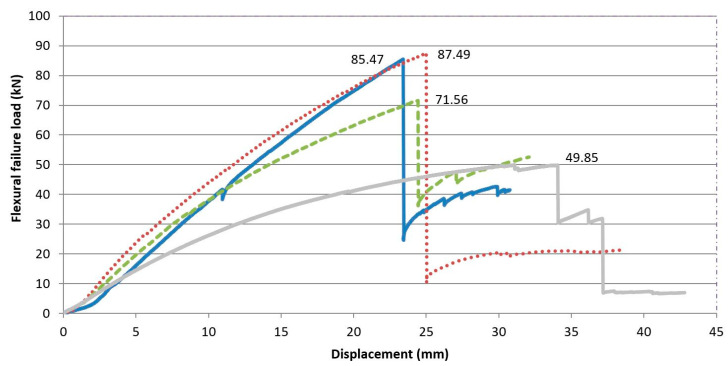
Full-scale test results.

The maximum load values under flexural failure load of the specimens, as well as their displacement may be seen in Table 6 and Figure 14. The values of the table are the result of the arithmetical average of the two test specimens of each constructive unit.

**Figure 15:**
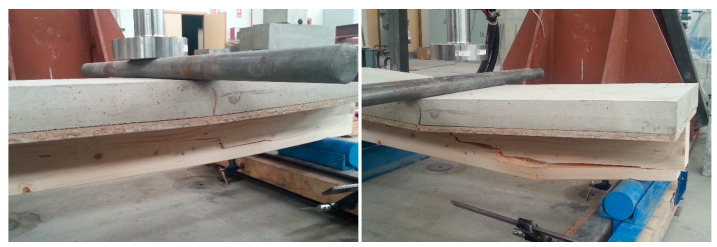
Final appearance of the specimens after the flexural test.

If the flexural behavior of the composite laminated wood-ecological mortar section is compared with the wooden joist, a higher maximum failure load can be observed for f the design model, with values that are 43.55% higher for the MLM mortar, and 71.45% higher for the MLC and MR mortars. This observation leads us to affirm that the mortar compression layer works through the connectors in unison with the wooden joist (Figure 15).

Likewise, if the deformations of the pieces are analyzed, a reduction of the displacement in the three composite laminated wood–mortar sections may be observed, with respect to the displacement of the wooden joist in isolation. The displacements, signs of impending breakage, for the three types of composite laminated wood–mortar section were very similar, at approximately 23–25 mm, while they reached 34 mm for the wooden joist in isolation. This behavior is explained by its lower inertia moment (I), and elasticity modulus (E).

### 4.2. Results Obtained by the Analytical Models

Applying the analytical model developed under Section 3.3, the maximum failure loads both of the laminated wood-ecological mortar section designs and of the wooden joist in isolation were obtained. In Table 7, the results are shown of the ultimate failure loads for the stress levels on both the lower fiber, *σ_i_* (shear), and on the upper fiber, *σ_s_* (compression).

In the same way as in the full-scale test, it was confirmed that both materials (wood and mortar) were working together as the failure load of the wooden joist—calculated with the analytical method—was lower than the failure load of the composite wood–mortar sections.

In contrast, the analytically calculated stress state of the wooden joist in isolation implies shear and compression forces on the lower and upper fibers of 15.80 MPa, which are below the tensile stress limits that are characteristic of wood under both flexion and compression (*f_mgk_* < 24 MPa).

The analytically tested stress state of the composite laminated wood–mortar section provided shear strengths of 15–19 MPa in the lower fibers, close to those obtained by the isolated wooden joist, but below the characteristic flexural stress (*f_mgk_* < 24 MPa). Moreover, the tensile stress under compression forces produced on the upper fiber, *σ_s_* (5–8 Mpa), were very much lower than the compression strengths of the mortars themselves (35 and 17 Mpa).

### 4.3. Results Obtained with the CYPE Program

The application of Cypecad v2019.e software [53] yielded the results of the response of the designed model, under eventual center-span loading. The most representative isovalue lines of the forces that are produced are shown so as to visualize the results of the laminated wood–mortar model.

**Figure 16:**
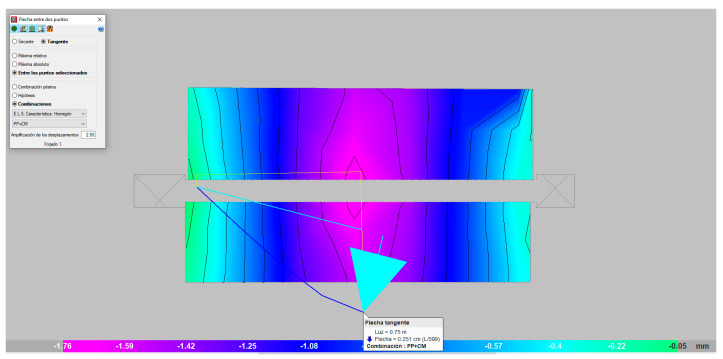
Deformation under the maximum load (mm).

In Figure 16, the isovalues corresponding to the deformation resulting from the action of the maximum failure load (mauve color) are shown, observing values between the two supports and at approximately 25 mm mid-span (blue color). The theoretical values were somewhat lower than those from the full-scale laboratory tests.

**Figure 17:**
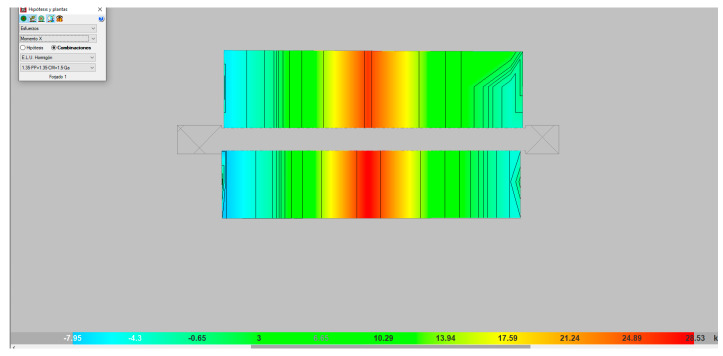
Moment on the *x*-axis (MPa).

In Figure 17, the values of the maximum moments obtained when applying the maximum failure load may be seen, expressed in isovalues (yellow–orange colors), which progressively diminished towards the supports (blue color). The values at those moments were similar to those obtained with analytical methods, such that the validity of the model may be affirmed.

### 4.4. Results Analysis and Commentaries

The increase in resistance of the composite section of the mortar beam was significantly higher compared to the isolated beam. The increase of the failure load that was observed in the full-scale tests implied strength increases of 43% for the MLM mortar type, 75% for the MLC mortar type, and 71% for the mixed MR mortar type, following the addition of the compressive layer acting in unison.

**Figure 18:**
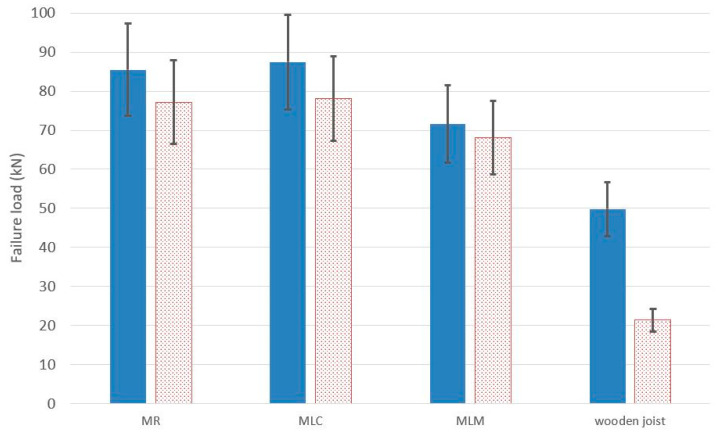
Full-scale test results vs. analytical results.

In Figure 18, the flexural failure load values from the full-scale test and the results of the analytical models are shown. The space or gap between both values is the safety margin.

As may be seen in Figure 18, the safety margin presents increases with respect to the analytical values, registering 10.71% in the case of reference mortar MR, 12.04% for mortar MLC, and 5.16% in the case of MLM.

The failure load of the composite laminated wood–mortar section, according to the analytical model, was lower than the results of the experimental tests. These results validated the mathematical models that were employed, because they confirmed that the safety margin was sufficient in response to the loads that were transferred.

In the case of the wooden joist in isolation, the safety margin was much higher, as the differences between the values of the maximum failure load in the full-scale tests, and those obtained from the analytical models were twice as high. This behavior can be justified, because a larger number of variables intervene in the calculation of the mechanical behavior of the composite section, such as the use of materials with different elasticity moduli, and the compatibility of the laminated wood–mortar interface working in unison.

## 5. Conclusions

A theoretical and practical study of the mechanical behavior of a construction unit designed with laminated wood and an ecological mortar has been conducted, for the restoration of wooden slabs within historic buildings. The following conclusions can be drawn from the analyses that were performed.

It has been confirmed that the incorporation of the mortar compression layer increased the strength of the slab, as opposed to the wooden joist in isolation at an average percentage of over 40%. It implies a very significant strength gain, because it will compensate possible drawbacks with the materials in use and mistakes that can occur in the construction process. It will even permit an increase of the load with no problems relating to the collapse of the construction unit.

The results obtained from the real-scale tests were very similar, both for the reference mortar (MR) and for the mortars designed with recycled aggregate, MLC and MLM. Nevertheless, the objective of the investigation is to design a constructive unit formed of laminated wooden joists and a mortar dosed with construction waste, for which reason mortars MLC and MLM complied with the required mechanical strength conditions. In addition, both mortars presented a very low density, approximately half of the reference mortar dosed with natural aggregates. This contributes a competitive advantage over traditional mortar, as it adds less weight to the existing structure, which is an essential factor in the rehabilitation of old buildings.

On the other hand, using mortars manufactured with recycled aggregate, both mixed and from concrete, is respectful towards the environment, thereby contributing to the circular economy of construction materials.

Positive safety margins have in all cases been tested, as the values from the full-scale experimental test models surpassed those of the theoretical analytical models. It may be affirmed that the full-scale laboratory results validated the analytical methodology that was applied.

From the results obtained with the analytical model and from the full-scale laboratory experimental test results, and their comparison, it was confirmed that the safety margin was greater for the wooden joist in isolation.

The tensile stress state of the joist in isolation, obtained with the mathematical model, showed that the forces of both shear and compression were below the characteristic stress limits under flexion and compression of the strength class of the wood that was employed, taken from Standard CTE-SE M (*f_mgk_* < 24 Mpa).

In the same way, the stress state of the composite laminated wood–mortar section from the analytical test methods gave shear strain limits in the lower fibers close to those obtained in the wooden joist in isolation, but very much lower compression forces in the upper fiber due to the compression of the mortar. This latter point demonstrates and corroborates that the experimental failures, in all cases, collapsed due to excessive stress on the lower fibers.

It may be added that the results from the analytical models were similar to the simulation process with CYPE software.

## 6. Patents

The mortar used in this research is protected by the Invention Patent: ES 2 629 064 B2 Mortero estructural aligerado con arcilla expandida y agregados con áridos reciclados [Structural lightweight mortar with expanded clay and aggregates with recycled fines]. The patent holder is the University of Burgos-Spain, and the inventors are the authors of this paper.

## Figures and Tables

**Table 1:** Mortar mixtures.

Sample	CEM I (gr)	Water (gr)	NA (gr)	ExC2/4 (gr)	ExC3/8 (gr)	RCA (gr)	RMA (gr)	Water/Cement Ratio
MR	500	330	2000	—	—	—	—	0.659
MLC	500	380	—	417.2	93.8	436.8	—	0.756
MLM	500	410	—	417.2	93.8	—	378.2	0.821

**Table 2:** Mortar characterizations.

	Test Method	MR	MLC	MLM
Water/cement ratio [37]	EN 1015-3 [41]	0.659	0.756	0.821
Bulk density of fresh mortar (kg/m^3^) [37]	EN 1015-6 [42]	2180	1280	1290
Air content (%) [37]	EN 1015-7 [43]	6.2	22	23
Dry bulk density of hardened mortar (kg/m^3^) [37]	EN 1015-10 [44]	2160	1170	1140
Flexural strength at 28 days (N/mm^2^) [37]	EN 1015-11 [45]	6.78	3.15	3.35
Compressive strength at 28 days (N/mm^2^) [37]	EN 1015-11 [45]	35.14	16.48	17.86
Water absorption by capillarity (kg/m^2^·min^0.5^) [38]	EN 1015-18 [46]	0.11	0.16	0.18
Surface abrasion resistance (mm) [38]	EN 14157 [47]	23.9	18.3	17.8
Mercury Intrusion Porosimetry (MIP) [38]	—	15.99	43.36	39.20
Durability: Freeze-thaw resistance (56 cycles) [38]	UNE CEN/TS 12390-9 EX [48]	Without alterations	Without alterations	Slight alterations

**Table 3:** Physical and mechanical properties of the 100 × 160 mm^2^ section of strength class GL24c.

Density (kg/m^3^)	Section (cm^2^)	Inertia Moment (cm^4^)	Strength Module W_i_ (cm^3^)	Characteristic Flexural Strength (*f_mgk_*) (N/mm^2^)	Characteristic Shear Strength (*f_vgk_*) (N/mm^2^)	Elasticity Modulus (E_m_) (kN/mm^2^)
350	160	3413	426	24	2.2	11.6

**Table 4:** Mechanical characteristics and properties of the wood–mortar composite section.

Sample	Y_G_ (cm)	Inertia Moment I_x_ (cm^4^)	Strength Distance Modulus (Y_G_) Lower Fiber W_i_ (cm^3^)	Strength Distance Modulus (Y_G_) Upper Fiber W_s_ (cm^3^)	E_m_/E_c_
MR	18.1	26,880	1517	4690	4.17
MLC-MLM	17.7	25,549	1445	4040	5.32

**Table 5:** Mortar dosages for the 70 L mixer.

Sample	CEM I (kg)	Water (l)	NA (kg)	ExC2/4 (kg)	ExC3/8 (kg)	RCA (kg)	RMA (kg)	Water/Cement Ratio
MR	25	16.48	100	—	—	—	—	0.659
MLC	25	18.90	—	20.86	4.69	21.83	—	0.756
MLM	25	20.53	—	20.86	4.69	—	18.91	0.821

**Table 6:** Results of the full-scale flexural test.

Sample	Flexural Failure Load (kN)		Displacement (mm)	
	Unit Value	Average Value	Unit Value	Average Value
MR	84.24	85.47	22.93	23.41
	86.70		23.89	
MLC	84.88	87.49	23.74	25.00
	90.10		26.26	
MLM	69.15	71.56	22.58	24.43
	73.97		26.28	
Wooden Joist	49.07	49.85	32.68	34.06
	50.63		35.44	

**Table 7:** Analytical results.

Sample	Analytical Failure Load (kN)	$\mathrm{Stress}\;\mathrm{on}\;\mathrm{Lower}\;\mathrm{Fiber},\;\sigma_{i},\;(\mathrm{MPa})\;*$	$\mathrm{Stress}\;\mathrm{on}\;\mathrm{Upper}\;\mathrm{Fiber},\;\sigma_{s},\;(\mathrm{MPa})\;**$
MR	77.20	15.81	−5.20
MLC	78.09	15.20	−5.03
MLM	68.05	19.03	−8.09
Wooden joist	21.39	15.80	−15.80

* values (+): shear; ** values (−): compression.

## References

[1] European Union. Opinion of the European Committee of the Regions (2015). Towards an Integrated Approach to Cultural Heritage for Europe. Official Journal of the European Union 195/04. https://eur-lex.europa.eu/legal-content/EN/TXT/PDF/?uri=OJ:C:2015:195:FULL&from=EN.

[2] Gobierno de España, Ministerio de Vivienda (2010). Integrated Urban Regeneration in the European Union Toledo Informal Ministerial Meeting on Urban Development. https://www.mitma.gob.es/recursos_mfom/pdf/2E3DD9C8-797E-4D50-9B7A-1F5CE0BEA9CA/111526/4_annexes_to_survey.pdf.

[3] Martín-Consuegra F., Alonso C., Frutos B. (2015). Integrated urban regeneration and the declaration of Toledo. Inf. Constr.

[4] Young E.H., Chan E.H. (2013). Evaluation for the conservation of historic buildings. Facilities.

[5] Cervero N., Agustín L. (2015). Urban Remodeling, Transformation and Renovation. Three ways of intervening on twentieth century Social Housing. Inf. Constr.

[6] Gospodini A. (2004). Urban morphology and place identity in European cities: Built heritage and innovative design. J. Urban Des..

[7] Pickard R. (2002). A comparative review of policy for the protection of the architectural heritage of Europe. Int. J. Herit. Stud..

[8] Calderoni C., De Matteis G., Giubileo C., Mazzolani F.M. (2006). Flexural and shear behaviour of ancient wooden beams: Experimental and theoretical evaluation. Eng. Struct..

[9] Branco J.M., Descamps T., Tsakanika E. (2018). Repair and strengthening of traditional timber roof and floor structures. Strengthening and Retrofitting of Existing Structures.

[10] Croce P., Beconcini M.L., Formichi P., Landi F., Cardella D. (2018). Fatigue behaviour of composite timber-concrete beams. Procedia Struct. Integr..

[11] Branco J.M., Tomasi R. (2014). Analysis and strengthening of timber floors and roofs. Structural Rehabilitation of Old Buildings.

[12] Trutalli D., Marchi L., Scotta R., Pozza L. Dynamic simulation of an irregular masonry building with different rehabilitation methods applied to timber floors. Proceedings of the 6th ECCOMAS Thematic Conference (COMPDYN 2017).

[13] Croci G. (1998). The Conservation and Structural Restoration of Architectural Heritage (Vol. 1).

[14] Marini A., Cominelli S., Zanotti C., Giuriani E. (2018). Improved natural hydraulic lime mortar slab for compatible retrofit of wooden floors in historical buildings. Constr. Build. Mater..

[15] Meda A., Riva P. (2001). Strengthening of wooden floors with high performance concrete slabs. Int. Z. Bauinstandsetz. Baudenkmalpflege.

[16] Faggiano B., Marzo A., Formisano A., Mazzolani F.M. (2009). Innovative steel connections for the retrofit of timber floors in ancient buildings: A numerical investigation. Comput. Struct..

[17] Garrido M., Correia J.R., Keller T., Branco F.A. (2015). Adhesively bonded connections between composite sandwich floor panels for building rehabilitation. Compos. Struct..

[18] Dias A. (2012). Analysis of the nonlinear behavior of timber-concrete connections. J. Struct. Eng..

[19] Gutkowski R.M., Brown K., Shigidi A., Natterer J. (2004). Investigation of notched composite wood–concrete connections. J. Struct. Eng..

[20] Richart F.E., Williams C.B. (1943). Tests of Composite Timber and Concrete Beams.

[21] Pincus G. (1969). Bonded wood-concrete T-beams. J. Struct. Div..

[22] Natterer J., Hamm J., Favre P. Composite wood-concrete floors for multi-story buildings. Proceedings of the International Wood Engineering Conference.

[23] Balogh J., Fragiacomo M., Gutkowski R., Fast R. (2008). Influence of repeated and sustained loading on the performance of layered wood–concrete composite beams. J. Struct. Eng..

[24] Clouston P., Bathon L.A., Schreyer A. (2005). Shear and bending performance of a novel wood–concrete composite system. J. Struct. Eng..

[25] Andersen M.S. (2007). An introductory note on the environmental economics of the circular economy. Sustain. Sci..

[26] Pomponi F., Moncaster A. (2017). Circular economy for the built environment: A research framework. J. Clean. Prod..

[27] Caviglia-Harris J.L., Kahn J.R., Green T. (2003). Demand-side policies for environmental protection and sustainable usage of renewable resources. Ecol. Econ..

[28] Sev A. (2009). How can the construction industry contribute to sustainable development? A conceptual framework. Sustain. Dev..

[29] Yılmaz M., Bakış A. (2015). Sustainability in construction sector. Procedia-Soc. Behav. Sci..

[30] Rodriguez Á., Manso J.M., Aragón Á., Gonzalez J.J. (2009). Strength and workability of masonry mortars manufactured with ladle furnace slag. Resour. Conserv. Recycl..

[31] Gómez-Rojo R., Alameda L., Rodríguez Á., Calderón V., Gutiérrez-González S. (2019). Characterization of polyurethane foam waste for reuse in eco-efficient building materials. Polymers.

[32] Saikia N., De Brito J. (2012). Use of plastic waste as aggregate in cement mortar and concrete preparation: A review. Constr. Build. Mater..

[33] Barriguete A.V., del Río Merino M., Sánchez E.A., Ramírez C.P., Arrebola C. (2018). Analysis of the feasibility of the use of CDW as a low-environmental-impact aggregate in conglomerates. Constr. Build. Mater..

[34] Agrela F., De Juan M.S., Ayuso J., Geraldes V.L., Jiménez J.R. (2011). Limiting properties in the characterisation of mixed recycled aggregates for use in the manufacture of concrete. Constr. Build. Mater..

[35] Pacheco-Torgal F., Jalali S. (2010). Reusing ceramic wastes in concrete. Constr. Build. Mater..

[36] Silva R.V., De Brito J., Dhir R.K. (2014). Properties and composition of recycled aggregates from construction and demolition waste suitable for concrete production. Constr. Build. Mater..

[37] Muñoz-Ruiperez C., Rodríguez A., Gutiérrez-González S., Calderón V. (2016). Lightweight masonry mortars made with expanded clay and recycled aggregates. Constr. Build. Mater..

[38] Muñoz-Ruiperez C., Rodríguez Á., Junco C., Fiol F., Calderón V. (2018). Durability of lightweight concrete made concurrently with waste aggregates and expanded clay. Struct. Concr..

[39] (2011). EN 413-1:2011 Masonry Cement—Part 1: Composition, Specifications and Conformity Criteria.

[40] (2014). EN 1097-6:2014 Tests for Mechanical and Physical Properties of Aggregates—Part 6: Determination of Particle Density and Water Absorption.

[41] EN 1015-3:2000 (2000). Methods of Test for Mortar for Masonry—Part 3: Determination of Consistence of Fresh Mortar (by Flow Table).

[42] (1999). EN 1015-6:1999 Methods of Test for Mortar for Masonry—Part 6: Determination of Bulk Density of Fresh Mortar.

[43] (1999). UNE-EN 1015-7:1999 Methods of Test for Mortar for Masonry—Part 7: Determination of Air Content of Fresh Mortar.

[44] (2007). EN 1015-10:2000/A1:2007 Methods of Test for Mortar for Masonry—Part 10: Determination of Dry Bulk Density of Hardened Mortar.

[45] (2007). EN 1015-11:2000/A1:2007 Methods of Test for Mortar for Masonry—Part 11: Determination of Flexural and Compressive Strength of Hardened Mortar.

[46] (2003). EN 1015-18:2003 Methods of Test for Mortar for Masonry—Part 18: Determination of Water Absorption Cefficient Due to Capillary Action of Hardened Mortar.

[47] (2005). EN 14157:2005 Natural Stone Test Methods—Determination of the Abrasion Resistance.

[48] (2008). UNE-CEN/TS 12390-9:2008 EX Testing Hardened Concrete—Part 9: Freeze-Thaw Resistance—Scaling.

[49] (2008). Instrucción de Hormigón Estructural, EHE-08.

[50] Software MTS Flex Test, GT.

[51] (2016). EN 1995-1-2:2016 Eurocode 5: Design of Timber Structures—Part 1–2: General—Structural Fire Design.

[52] (2019). Código Técnico de la Edificación—Documento Básico DB-SE-M—Seguridad Estructura Madera.

[53] Thouless M.D. (2018). Shear forces, root rotations, phase angles and delamination of layered materials. Eng. Fract. Mech..

[54] Software CYPECAD v 2019.

